# Factors Associated With Hearing Aid Use Among Medicare Beneficiaries

**DOI:** 10.1093/geroni/igab021

**Published:** 2021-06-23

**Authors:** Lama Assi, Nicholas S Reed, Carrie L Nieman, Amber Willink

**Affiliations:** 1 Cochlear Center for Hearing and Public Health, Johns Hopkins Bloomberg School of Public Health, Baltimore, Maryland, USA; 2 Department of Epidemiology, Johns Hopkins Bloomberg School of Public Health, Baltimore, Maryland, USA; 3 Department of Otolaryngology–Head and Neck Surgery, Johns Hopkins School of Medicine, Baltimore, Maryland, USA; 4 Menzies Centre for Health Policy and Economics, University of Sydney, Camperdown, Australia

**Keywords:** Access to care, Health services research, Hearing care, Hearing loss

## Abstract

**Background and Objectives:**

In the United States, up to two-thirds of older adults have hearing loss. Untreated hearing loss can have significant health outcomes, yet less than 20% of adults with hearing loss use hearing aids. In this study, we examined potential factors associated with hearing aid use, including detailed measures of health status, access to care, patient engagement, and technology use, in a nationally representative sample of Medicare beneficiaries.

**Research Design and Methods:**

Cross-sectional study using the 2017 Medicare Current Beneficiary Survey. Participants with self-reported hearing loss were included. The primary outcome was hearing aid use. Factors potentially associated with hearing aid use included: sociodemographics, health determinants, access to care, patient activation, and technology access/use.

**Results:**

Overall, 5,146 participants were included. Of them, 27% reported using hearing aids. In a multivariable logistic regression model, predisposing factors associated with greater odds of hearing aid use included older age, identifying as a man, identifying as White, having completed college, having 3 or more chronic conditions, having dementia, not having trouble seeing, not having limitations in activities of daily living, having moderate relative to low information-seeking scores, and having a personal computer at home (range of odds ratios [ORs]: 1.22–4.46). Enabling factors associated with greater odds of hearing aid use included higher income, living alone relative to living with family members other than a spouse, and having a usual source of care (range of ORs: 1.43–1.54).

**Discussion and Implications:**

In addition to addressing previously identified factors associated with hearing aid use, improving access to health care, technology, and information about hearing aids may improve the uptake of hearing aids. These findings help further inform our understanding on how to address low treatment levels of hearing loss in the community by identifying new populations to target and potentially modifiable risk factors for hearing aid use.

**Translational Significance:** Health care behaviors such as having a routine place for health care and seeking health information may translate into hearing aid uptake among those with hearing loss. Medical providers who are more forthcoming with information on hearing loss and treatments may help bridge this access gap.

## Background and Objectives

Hearing loss affects up to two-thirds of older adults in the United States (Goman & Lin, 2016). It is associated with significant health outcomes including social isolation, and increased risk of falls, dementia, hospitalizations, health care spending, and death (Deal et al., 2017; Genther et al., 2015; Reed et al., 2019; Shukla et al., 2020; Viljanen et al., 2009). Hearing aid use could potentially modify these associations as a protective factor. Trials have shown that it improves quality of life and communication function (Ferguson et al., 2017; Mulrow et al., 1990). Moreover, recent observational studies suggest adults with hearing loss who use hearing aids have a reduced risk of dementia, falls, and hospitalizations relative to those who do not use them (Mahmoudi et al., 2018, 2019).

Despite this, less than 20% of adults with hearing loss in the United States use hearing aids (Chien & Lin, 2012). A key barrier to accessing hearing aids is affordability. However, in the United Kingdom, where the full cost of hearing aids is covered by the National Health Service, a third of patients fit with hearing aids do not use them regularly (Aazh et al., 2015). Therefore, hearing aid use as a health behavior is more complex than a question of affordability alone. Previous work suggests multiple factors associated with hearing aid use include income, education, race/ethnicity, health status, technology use, stigma, and family/friend support (Bainbridge & Ramachandran, 2014; McKee et al., 2019; Nieman et al., 2016; Tahden et al., 2018).

Patient engagement and health-seeking behaviors may be key factors that fuel health behaviors (Hibbard & Greene, 2013; Spatz et al., 2010). However, few studies have explored these factors in association with hearing aid use specifically. In this study, we examine potential factors associated with hearing aid use, including detailed measures of health status, health care access, patient engagement, and technology use in the 2017 Medicare Current Beneficiary Study (MCBS), a nationally representative sample of Medicare beneficiaries. A more robust understanding of the factors behind hearing aid use could help target those who are least likely to access hearing aids.

## Research Design and Methods

The MCBS is an ongoing survey of the Medicare program’s impact on beneficiaries. Analyses were limited to participants who reported hearing difficulty, defined through their use of a hearing aid or not using a hearing aid but having a little or a lot of trouble hearing. Participants were asked: “Do you use a hearing aid? Yes, no,” followed by: “Which statement best describes your hearing [with a hearing aid]? No trouble, a little trouble, a lot of trouble.”

The primary outcome was self-reported hearing aid use (“Do you use a hearing aid? Yes, no”). Covariates were identified using the Andersen–Aday Behavioral Model of Health Services Use framework, which describes drivers of health care utilization fitting into one of the following categories: predisposing characteristics, enabling factors, and perceived need (Andersen, 1995). Predisposing characteristics included age, gender, race/ethnicity, education, number of chronic conditions, depression, self-reported diagnosis of dementia, trouble with vision, limitations in activities of daily living (ADLs), having a helper for a functional impairment, information-seeking scores, and technology use (having a personal computer, using the Internet). Enabling factors included income relative to the federal poverty line, urban status of the area of residence, living arrangement, health insurance with hearing aid coverage, and having a usual source of care. Only participants with the perceived need factor (defined as having trouble hearing or using a hearing aid) were included.

A multivariable logistic regression model examined the association of hearing aid use with the predisposing characteristics and enabling factors. ​Coefficients were exponentiated to odds ratios (ORs) for ease of interpretation. To consistently report on factors associated with greater (rather than lower) odds of hearing aid use, ORs below 1.00 were converted to their inverse and categories were reversed when describing the results in the text. Survey weights were used to account for the MCBS sampling design and survey nonresponse. Analyses were conducted using Stata/SE (Version 14). The Institutional Review Board of NORC at the University of Chicago approved the MCBS study.

## Results

In total, 5,146 participants were included, representing a weighted sample of 21,341,218 Medicare beneficiaries with self-reported hearing loss. Overall, 42.2% were 75 years and older, 51.9% were women, and 86.9% identified as White. Less than a third (27.0%) reported using hearing aids. A greater proportion of participants who used hearing aids were 75 years and older, men, and identified as White (Table 1).

**Table 1.** Characteristics of the Medicare Population With Hearing Loss by Hearing Aid Use in Weighted Percentages, 2017

	Total population with hearing loss	Uses a hearing aid among those with hearing loss		
Characteristics		No	Yes	*p* Value
Sample size (unweighted)	5,146	3,559	1,587	
Population size (weighted)	21,341,218	15,597,951	5,743,267	
Population distribution (row %)	100%	73%	27%	
	Column weighted percentages			
Age, in years				<.001
<65	12.4%	15.6%	3.8%	
65–74	45.4%	48.2%	37.9%	
75–84	29.6%	27.4%	35.4%	
85+	12.6%	8.8%	22.9%	
Men	51.9%	48.8%	60.2%	<.001
Race				<.001
White	86.9%	85.2%	91.3%	
Black	7.3%	8.6%	3.7%	
Other	5.8%	6.2%	4.9%	
Educational attainment				<.001
Less than high school	13.2%	14.5%	9.7%	
High school graduate	51.4%	52.4%	48.8%	
Completed college	35.4%	33.2%	41.5%	
Number of chronic conditions				.06
None	6.7%	7.0%	6.0%	
1–2	36.1%	36.6%	34.8%	
3–5	45.1%	44.2%	47.6%	
6+	12.1%	12.3%	11.6%	
Has depression	12.2%	14.1%	7.2%	<.001
Has dementia	2.5%	2.3%	3.2%	<.05
Has trouble with vision	42.4%	45.4%	33.9%	<.001
Number of limitations in ADLs				<.001
0 limitations in ADLs	69.3%	67.4%	74.4%	
1 limitation in ADLs	14.7%	15.2%	13.2%	
2+ limitations in ADLs	16.1%	17.4%	12.4%	
Has a helper	31.0%	32.4%	27.3%	<.001
Information seeking				<.001
Low information seeking	29.1%	30.3%	25.7%	
Moderate information seeking	38.4%	37.8%	40.1%	
High information seeking	32.5%	31.8%	34.2%	
Has a personal computer at home	72.3%	70.6%	77.3%	<.001
Ever use the Internet to get information	65.2%	64.7%	66.7%	.09
Income poverty ratio Medicare threshold				<.001
<100% of the FPL	10.5%	12.3%	5.7%	
100–149% of the FPL	12.8%	14.1%	9.2%	
150–199% of the FPL	10.7%	11.2%	9.5%	
200–399% of the FPL	29.9%	29.0%	32.3%	
≥400% of the FPL	36.1%	33.4%	43.4%	
Urban area of residence	76.6%	76.3%	77.0%	.61
Living arrangement				<.001
Alone	28.9%	28.9%	28.6%	
Spouse	53.8%	51.7%	59.7%	
Children/family	10.7%	12.0%	7.3%	
Other	6.6%	7.4%	4.5%	
Hearing aid insurance coverage	10.7%	11.1%	9.5%	.05
Has a usual source of care	93.4%	92.4%	95.9%	<.001

*Notes*: ADL = activities of daily living; FPL = federal poverty level.

### Predisposing Factors

Greater odds of hearing aid use were associated with being between the ages 65 and 74 years relative to younger than 65 years (OR = 2.00, 95% confidence interval [CI] = 1.43, 2.85), and being 75 years or older relative to being between the ages 65 and 74 years (75–84 years vs 65–74 years, OR = 1.79, 95% CI = 1.60, 1.99; 85 years and older vs 65–74 years, OR = 4.46, 95% CI = 3.85, 5.18), identifying as a man relative to a woman (OR = 1.59, 95% CI = 1.41, 1.79), identifying as White relative to Black (OR = 1.52, 95% CI = 1.08, 2.13), having completed college relative to not having completed high school (OR = 1.25, 95% CI = 1.03, 1.51), having three or more chronic conditions (3–5 chronic conditions vs no chronic conditions, OR = 1.35, 95% CI = 1.10, 1.66; six or more chronic conditions vs no chronic conditions, OR = 1.53, 95% CI = 1.20, 1.94), having dementia relative to not (OR = 1.53, 95% CI = 1.16, 2.03), not having trouble with vision relative to having trouble (OR = 1.30, 95% CI = 1.16, 1.43), not having limitations in ADLs relative to having one limitation (OR = 1.22, 95% CI = 1.02, 1.45), having moderate information-seeking scores relative to low scores (OR = 1.22, 95% CI = 1.08, 1.39), and having a personal computer at home relative to not (OR = 1.33, 95% CI = 1.13, 1.56; Table 2). Having depression, having a helper, and using the Internet to get information were not associated with hearing aid use.

**Table 2.** Adjusted ORs of Hearing Aid Use by Predisposing Characteristics and Enabling Factors Among Medicare Beneficiaries With Hearing Loss, 2017

Variables	OR (95% CI)
Predisposing characteristics	
Age, in years (ref: 65–74 years)	
<65	0.50*** (0.35–0.70)
75–84	1.79*** (1.60–1.99)
85+	4.46*** (3.85–5.18)
Women (ref: men)	0.63*** (0.56–0.71)
Race (ref: White)	
Black	0.66* (0.47–0.93)
Other	0.92 (0.73–1.16)
Education (ref: less than high school)	
High school graduate	1.14 (0.96–1.34)
Completed college	1.25* (1.03–1.51)
Number of chronic conditions (ref: none)	
1–2	1.21 (0.98–1.50)
3–5	1.35** (1.10–1.66)
6+	1.53*** (1.20–1.94)
Depression (ref: no depression)	0.86 (0.69–1.07)
Dementia (ref: no dementia)	1.53** (1.16–2.03)
Trouble with vision (ref: no trouble with vision)	0.77*** (0.70–0.86)
Number of limitations in ADLs (ref: 0 limitations in ADLs)	
1 limitation in ADLs	0.82* (0.69–0.98)
2+ limitations in ADLs	0.92 (0.76–1.13)
Has a helper (ref: no helper)	1.08 (0.94–1.26)
Information seeking (ref: moderate information seeking)	
Low information seeking	0.82** (0.72–0.93)
High information seeking	1.09 (0.97–1.23)
Has a personal computer at home (ref: no)	1.33*** (1.13–1.56)
Ever use the Internet to get information (ref: no)	0.97 (0.86–1.10)
Enabling factors	
Income poverty ratio Medicare threshold (ref: <100% of the FPL)	
100–149% of the FPL	1.05 (0.80–1.37)
150–199% of the FPL	1.23 (0.94–1.59)
200–399% of the FPL	1.45** (1.15–1.83)
400%+ of the FPL	1.53*** (1.21–1.94)
Urban area of residence (ref: rural)	0.91 (0.78–1.07)
Living arrangement (ref: alone)	
Spouse	1.05 (0.89–1.25)
Children/family	0.70*** (0.58–0.85)
Other	0.86 (0.62–1.20)
Hearing aid insurance coverage (ref: no hearing aid coverage)	1.04 (0.89–1.22)
No usual source of care (ref: yes)	0.65*** (0.51–0.81)

*Notes*: ADL = activities of daily living; CI = confidence interval; FPL = federal poverty level; OR = odds ratio; ref = reference.

*p < .05. **p < .01. ***p < .001.

### Enabling Factors

Higher income (income to poverty ratio 200%–399% vs less than 100%, OR = 1.45, 95% CI = 1.15, 1.83; income to poverty ratio greater than 400% vs less than 100%, OR = 1.53, 95% CI = 1.21,1.94), living alone relative to living primarily with children/other family members (OR = 1.43, 95% CI = 1.18, 1.72), and having a usual source of care relative to not having one (OR = 1.54, 95% CI = 1.23, 1.96) were associated with higher odds of hearing aid use. Urban area of residence and having hearing insurance coverage were not associated with hearing aid use (Table 2).

## Discussion and Implications

In a nationally representative sample of Medicare beneficiaries with hearing loss, only 27% reported using hearing aids. In addition to previously identified factors associated with hearing aid use, we found that having access to technology, a usual source of care and moderate relative to low health information-seeking scores were associated with greater odds of use.

Having a usual source of care has been shown to have a positive effect on health care utilization, including preventive care (e.g., immunization, cancer screening; Kim et al., 2012). Our analysis showed that these benefits may extend to addressing hearing loss as those with a usual source of care were more likely to use hearing aids than those without. A study among U.S. residents reported that individuals’ type of routine place of care was not associated with hearing aid use; however, having a routine place of care relative to not having one was not assessed (Bainbridge & Ramachandran, 2014). This supports the notion that compared to no usual source of care, a usual source of care provider, regardless of type, may better identify needs as they develop over time.

However, screening for hearing loss is not uniformly conducted or addressed by providers (McKee et al., 2019). Apart from the Veterans Health Administration hospital system where hearing screening is mandated, most primary care providers do not screen for hearing loss (Zazove et al., 2017). Therefore, it may not be enough to have a usual source of care. Individuals may find themselves having to actively seek hearing care or mention hearing problems to their primary care providers, which could explain why those with moderate information-seeking scores were more likely than those with low scores to use hearing aids. These findings suggest that those who use hearing aids may be fundamentally different than those who do not in how they engage with the health system. As a result, the better health care utilization patterns found among hearing aid users relative to nonusers in observational studies (Mahmoudi et al., 2018) may reflect differences in general patterns of health care use rather than the impact of treating hearing loss, highlighting the need for interventional studies.

The cost of hearing aids has been proposed as a significant barrier to hearing aid use with studies showing marked differences in hearing aid utilization by income (Willink et al., 2020). The impact of the cost of hearing aids to an individual may be dampened by insurance coverage. In our study, having hearing aid coverage was not associated with hearing aid use. This could be because hearing coverage under Medicare Advantage plans is insufficient to support the purchase of hearing aids, as beneficiaries with these plans still pay over 70% of the total spending on hearing care (Willink et al., 2020). It could also be that access to care measures and health-seeking behaviors, beyond insurance coverage, may be more important in determining hearing aid use, consistent with the low prevalence of regular hearing aid use in the United Kingdom (Aazh et al., 2015).

We also found that indicators of poorer health (comorbidities, dementia) were associated with greater odds of hearing aid use, but functional impairments (trouble seeing, limitations in ADLs) were associated with lower odds of use. This is consistent with general patterns of health care use, whereby disability is generally associated with access barriers (Kaye, 2019), and multimorbidity with increased health care use (Lehnert et al., 2011). Therefore, general health care utilization patterns could be translating into hearing aid use patterns. Importantly, addressing the access to care barriers faced by those with vision loss and disabilities may help improve the uptake of hearing aids. Facilitating hearing aid usage for those with dual sensory impairment (concurrent hearing and vision impairments) may require specialized care including considerations during prefitting, fitting, and postfitting (Kricos, 2007; Vreeken et al., 2014).

In our study, access to technology (having a personal computer) was associated with greater odds of hearing aid use, but technology use (using the Internet to get information) was not. Prior studies have demonstrated that people with higher technology commitment scores, defined as better technology competence, acceptance, and control, were more likely to use hearing aids (Tahden et al., 2018). Technology adoption by older adults, specifically, may be driven by early experiences, self-efficacy, and cognitive abilities (Mitzner et al., 2019). In our study, technology use was limited to one question and may not fully capture these concepts. The association between access to technology and hearing aid use may suggest that those who use hearing aids are more familiar with technology, which could be beneficial with the growing innovations to hearing aid technology (Hoppe & Hesse, 2017).

Consistent with previous studies, we found that older age, identifying as White, having higher educational attainment, and higher income were associated with hearing aid use, and living in an urban versus rural area was not associated with hearing aid use (Bainbridge & Ramachandran, 2014; McKee et al., 2019; Nieman et al., 2016). We also reported that living alone rather than living with children or family without a spouse was associated with greater odds of hearing aid use. However, support from friends and family has been shown to facilitate obtaining and using hearing aids in other cohorts (McKee et al., 2019). Of note, living arrangements that do not include a spouse have been associated with health disadvantages (Hughes & Waite, 2002), and this could include lack of hearing aid use.

Our study has limitations that should be taken into account when interpreting the results. First, our outcome measure does not distinguish between regular and nonregular hearing aid use, or hearing aid ownership. Second, only those with self-reported hearing trouble or hearing aid use were included in the study; thus, results may not be generalizable to those who do not know that they could benefit from hearing aids. Finally, temporality between the variables assessed and hearing aid use cannot be established based on these cross-sectional data.

In conclusion, in addition to the previously reported factors associated with hearing aid use, we found that technology use and health care behaviors such as information seeking and having a usual source of care may influence hearing aid uptake. These findings could help better target those who may benefit from interventions to improve hearing aid use.
